# Using perceptual tasks to selectively measure magnocellular and parvocellular performance: Rationale and a user’s guide

**DOI:** 10.3758/s13423-020-01874-w

**Published:** 2021-03-19

**Authors:** Mark Edwards, Stephanie C. Goodhew, David R. Badcock

**Affiliations:** 1 Research School of Psychology, The Australian National University, Canberra, Australia; 2 School of Psychological Sciences, The University of Western Australia, Crawley, Australia

**Keywords:** Visual perception, Magnocellular, Parvocellular, Dorsal, Ventral, Visual pathways

## Abstract

The visual system uses parallel pathways to process information. However, an ongoing debate centers on the extent to which the pathways from the retina, via the Lateral Geniculate nucleus to the visual cortex, process distinct aspects of the visual scene and, if they do, can stimuli in the laboratory be used to selectively drive them. These questions are important for a number of reasons, including that some pathologies are thought to be associated with impaired functioning of one of these pathways and certain cognitive functions have been preferentially linked to specific pathways. Here we examine the two main pathways that have been the focus of this debate: the magnocellular and parvocellular pathways. Specifically, we review the results of electrophysiological and lesion studies that have investigated their properties and conclude that while there is substantial overlap in the type of information that they process, it is possible to identify aspects of visual information that are predominantly processed by either the magnocellular or parvocellular pathway. We then discuss the types of visual stimuli that can be used to preferentially drive these pathways.

## Introduction

The concept of parallel pathways in visual processing, which preferentially extract particular types of information, has been influential, but controversial. From the retina onwards, an important distinction has been made between the magnocellular (M) and parvocellular (P) pathways, based on differences in their morphology and connectivity (Kaplan, 2004, 2012; Nassi & Callaway, 2009). The extent to which the tuning properties of cells in these pathways differ, and hence the degree of specialization in the type of visual information they process has been debated. The views range from them being specialized for processing largely distinct types of information (Livingstone & Hubel, 1988; Ungerleider & Mishkin, 1982) to there being no functional differences between them for stimuli that are above luminance-contrast detection thresholds, with only a possible difference at luminance-contrast detection thresholds (Skottun, 2000, 2015, 2016). An intermediate view is that though there is substantial overlap in the tuning properties, there are significant differences that lead to them extending the sensitivity of the visual system along different dimensions, and hence it is possible to selectively drive them, but care needs to be taken in order to do so (DeYoe & Van Essen, 1988; Kaplan, 2012; Schiller, Logothetis, & Charles, 1990b). These disparate views have led to significant debate in the literature, for example (Butler et al., 2007; Dhar, Been, Minderaa, & Althaus, 2010; Lalor & Foxe, 2010; Ogmen, Purushothaman, & Breitmeyer, 2008).

The extent to which these pathways are tuned to different aspects of the visual scene, and hence our ability to tailor visual stimuli to selectively stimulate these pathways, has emerged as an important issue for two main reasons. The first is due to the proposal that certain pathologies result from impaired functioning of particular pathways (for a review, see Grinter, Maybery, & Badcock, 2010). For example, dyslexia and schizophrenia have been linked to impairments in the M pathway. This has led to attempts to compare the functioning of that pathway in those groups with neurotypical controls (Badcock & Lovegrove, 1981; Butler et al., 2005; Gori, Seitz, Ronconi, Franceschini, & Facoetti, 2016; Kim, Wylie, Pasternak, Butler, & Javitt, 2006; Laycock & Crewther, 2008; Lovegrove, Bowling, Badcock, & Blackwood, 1980; Schechter et al., 2005). The second reason is that, in cognitive psychology, attempts have been made to link certain cognitive functions to specific pathways. For example, it has been proposed that attentional modulation may have differing effects on the M and P pathways (Goodhew, Shen, & Edwards, 2016; Vidyasagar, 2004; Yeshurun & Carrasco, 1998; Yeshurun & Levy, 2003; Yeshurun & Sabo, 2012; though also see Lawrence, Edwards, & Goodhew, 2020), and that the proximity of the hands can alter the relative contribution of the two pathways to visual perception (Bush & Vecera, 2014; Goodhew, Edwards, Ferber, & Pratt, 2015; Gozli, West, & Pratt, 2012). Note that the koniocellular pathway is another important pathway that project from the dLGN (Kaplan, 2012). For an overview of the properties of this pathway see Kaplan (Kaplan, 2012 and Martin & Solomon, 2019).

The answer to the question of whether we can selectively drive the M and P cells via particular laboratory stimuli and tasks depends on how different their tuning properties are. That is, do M and P cells truly process different aspects of the visual scene such that different perceptual tasks can dissociate their function from one another? In this review, we will examine the evidence relevant to this question, focusing on the anatomical, electrophysiological, and lesion studies in relation to the M and P pathways. Based on this review, we will argue that it is possible to selectively stimulate them via laboratory stimuli and tasks, and thereby measure their distinct contribution to visual perception. We will describe what these differences are and, based on these differences, explain how to optimize visual stimuli to selectively stimulate the cells. This review is aimed at a general readership, including those researchers who predominantly conduct cognitive studies, and who therefore may not be familiar with some of the technical aspects of these issues. Accordingly, we will spend time explaining some of these aspects in order to clarify these issues and resolve some of the common misunderstandings in this area.

## Anatomical differences

In the primate retina, there are about 20 distinct ganglion-cell populations that project in parallel to other parts of the brain (Dacey, 2004). The two ganglion cell types that are important here are the parasol and midget cells that selectively project to the M and P layers of the dorsal lateral geniculate nucleus (dLGN), respectively. The parasol cells constitute around 10% of the cells that project to the dLGN and the midget cells around 60% to 70% (depending on the species). A third important retinal cell type is bistratified cells, which form at least part of the input to the koniocellular cells in the dLGN and constitute around 8% of the cells that project to the dLGN (Dacey & Petersen, 1992; Kaplan, 2012; Schiller & Logothetis, 1990). In the dLGN, the M and P cells are arranged in distinct layers, with the lower two layers comprising M cells (one layer for each eye) and the upper four comprising P cells (two layers for each eye). These cells were originally distinguished by their morphology, with M cells being larger than P cells (hence their names, with “magno” in this context meaning large, and “parvo” meaning small). K cells are found predominantly between the M and P layers. The relative proportion of these cells in the dLGN largely mirrors the relative numbers of their input cells (parasol and midget cells), with roughly 10% being M and 80% being P cells, and K cells making up the most of the remaining 10% of cells (Dacey, 2004; Kaplan, 2004; Merigan & Maunsell, 1993; Nassi & Callaway, 2009).

The segregation of the M and P cells continues up to their projection layers in the primary visual cortex (V1), with M cells projecting to layers 4Cα and P cells to 4Cβ. It was originally thought that these pathways remained segregated at higher levels in the cortex, with the M and P cells then selectively projecting pathways projecting through the dorsal and ventral brain regions, respectively (Maunsell, 1987). However, while dorsal and ventral pathways receive most of their input from the M and P pathways respectively, there is substantial cross talk (Maunsell & Newsome, 1987). This occurs even at the input level to V1, with collaterals of both the M and P input projecting to Layer 6, which then feeds back to the dLGN, creating the ability for the cortex to modify its own input (Ferrera, Nealey, & Maunsell, 1992; Kaplan, 2004; Livingstone & Hubel, 1987; Marrocco, McClurkin, & Young, 1982; Nassi & Callaway, 2009; Sincich, Park, Wohlgemuth, & Horton, 2004). While there is not complete segregation of the M and P pathways beyond V1, there does seem to be a high degree of functional modularity that occurs from V1 to V2 and then onto higher cortical areas like V4 and V5/MT. Specifically, distinct regions in V1 and V2 can be identified due to their differential density of the enzyme cytochrome oxidase (CO—which is a marker for metabolic activity; Takahata, 2016) and they receive, at least dominant, if not selective, input from the M or P cells. In V1, regions of high CO concentration are arranged in blobs with the interblob regions having low concentrations of CO, while in V2 the regions of high concentration are arranged in thin and thick stripes, with interstripe regions of low density. The P cells preferentially project to the blob and interblob regions which then project to the thin and interstripe regions, respectively, and then onto subregions of V4 and the posterior inferotemporal ventral (PITv) area. The M cells project to the thick stripes in V2 and then onto V5/MT (middle temporal area) and other areas in the dorsal pathway (DeYoe & Van Essen, 1988; Felleman, Xiao, & McClendon, 1997; Lu & Roe, 2008; Roe & Ts'o, 1995; Van Essen & Gallant, 1994; Xiao & Felleman, 2004; Xiao, Wang, & Felleman, 2003; Xiao, Zych, & Felleman, 1999). We will discuss the functional specialization of these pathways, and specifically, the two P cell dominated pathways, in the section Unique Roles of the M and P Pathways. Note also that small populations of cells, predominantly K cells but also M and P cells, project directly to areas beyond V1, specifically V5/MT, V4, and V2 (Bullier & Kennedy, 1983; Sincich et al., 2004; Yukie & Iwai, 1981).

The concept that the M and P pathways would be involved in processing different types of visual information had face validity, given their segregation up to the cortical level, and the initial belief that they remained segmented at the cortical level. What would be the functional reason for their demarcation if they were not processing, at least to some extent, different types of information? The two main techniques that have been used to investigate their properties are electrophysiological and lesion studies, both in nonhuman primates.

## Electrophysiology

One of the first things to consider with electrophysiological studies that have recorded the activity of cells in the M or P layers in the dLGN of nonhuman primates is that they have sampled a very small proportion of the total number of cells. In a stereological study using postmortem human brain samples from three groups (subjects who had schizophrenia, a heterogeneous group who had mood disorders, and neurotypical comparison subjects), it was estimated that a healthy human dLGN contains around 2 million neurons (Dorph-Petersen et al., 2009). As we will see, the highest number of cells recorded from in electrophysiological studies (in nonhuman primates) are around 3–4 hundred. While this represents a substantial amount of recording time, it is still only around 0.0002% of the total number of cells (in a human). Note that in all of the electrophysiological studies discussed below, nonhuman primates were tested. This very sparse sampling of cells may create issues in generalizing the results of these studies to the functioning of a human brain.

### Contrast sensitivity

Both M and P cells at the retinal and dLGN levels have spatially opponent receptive fields. That means that it is possible to map out regions in their receptive field that respond in either an excitatory or inhibitory manner to the presence of light in those regions. These excitatory and inhibitory regions are arranged in a roughly circular manner and there are two types of cells based on how they are arranged. “On” cells have an excitatory center and an inhibitory surround, and “off” cells have an inhibitory center and an excitatory surround. At the dLGN level, these excitatory and inhibitory regions are balanced, which means that the cells do not respond to stimulation by uniform luminance fields. Instead, they respond to luminance differences (i.e., luminance contrast) between the center and surround regions. “On” cells respond when a bright dot of light is presented in their center, and “off” cells respond when a dark dot is presented in the center. A higher contrast stimulus means that the luminance difference between the stimulus and the background is increased, so, for example, the luminance of a bright dot is increased, while the background luminance remains constant. The response of a cell varies as a function of the luminance contrast of the stimulus, and this can be plotted with the slope of that graph being referred to as the contrast gain of the cell. The greater the contrast gain, the greater the increase in the cell’s response with increasing contrast. M cells have both a greater response and a higher contrast gain at low contrasts (Derrington & Lennie, 1984; Kaplan & Shapley, 1982). For example, Kaplan and Shapley (1982) found that to elicit a response of five impulses/s, M cells in their sample only required a contrast of 1.2%, while P cells required a contrast of 9.1%.

The high contrast gain of M cells at low contrasts is often taken to mean that their responses saturate at higher (around 20%) contrasts (Derrington & Lennie, 1984). However, at least at the retinal level, while the contrast gain of M (parasol) cells increases less rapidly after about 20% contrast, they still have a contrast gain that is similar to that of P (midget) cells (Kaplan & Shapley, 1986). This finding is consistent with the finding from a behavioral study that used high-speed motion stimuli and hence was mediated by motion-sensitive cells (that are thought to receive dominant input from M cells). Differential performance was obtained to differences in stimulus contrast at high (greater than 60%) contrast levels (Edwards, Badcock, & Nishida, 1996). These findings mean that M cells will provide larger responses to low-contrast stimuli, as long as the spatial and temporal properties of the stimuli fall within the sensitivity range of the M cells.

### Spatial-tuning properties

**Fig. 1:**
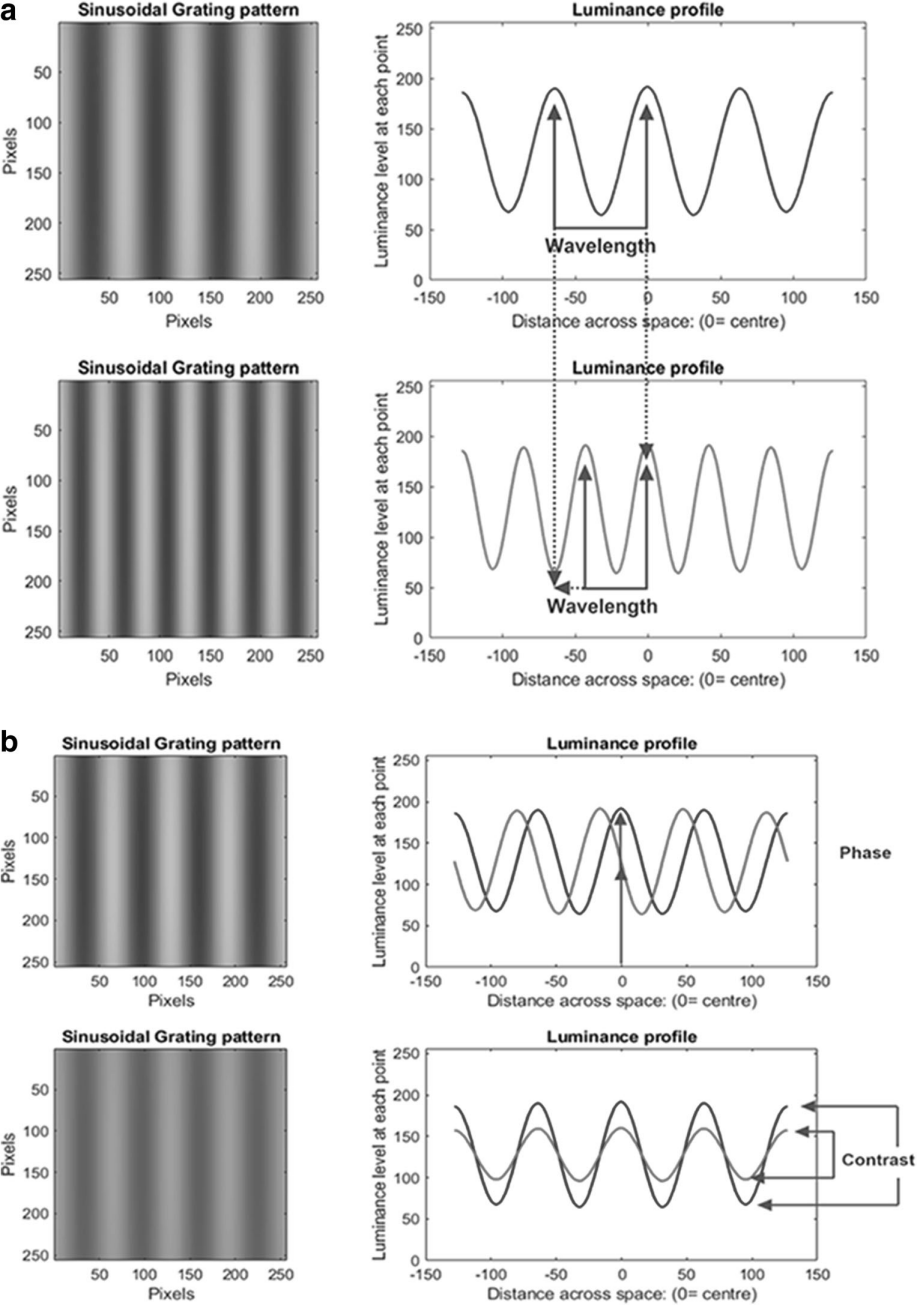
Demonstrations of the building blocks used in spatial frequency analysis. The left-hand images depict the actual sinewave gratings. **a** Demonstrates variation in spatial frequency (1/wavelength in degrees). The upper image has a longer wavelength and hence represents a lower spatial frequency. **b** Demonstrates variation in phase and contrast

Spatial tuning refers to the sensitivity of the cells to how the luminance in the image changes across space. Determining the sensitivity of the cells to *spatial frequency* is the standard way to measure their ability to resolve this spatial information. It is based on the notion of Fourier analysis, which states that any image (function) can be represented by a series of sinewaves (Bracewell, 1978). In this context, the image is the variation in luminance across space, and the sinewaves are bars of light whose luminance varies from light to dark in a sinewave manner across space. These sinewaves vary in their spatial frequency, contrast, and phase. Spatial frequency refers to the scale of the sinewave—that is, how rapidly the sinewave cycles between light and dark. This is the inverse of the wavelength of the sinewave, which is the distance between luminance peaks (i.e., the size of one sinewave cycle). Contrast refers to the magnitude of the luminance modulation (i.e., how much the peaks and troughs vary from the mean [background] luminance). Phase refers to the starting point of the sinewave—that is, does it start, for example at a peak or a trough. Refer to Fig 1. The spatial-frequency approach entails determining how sensitive a particular cell (or person) is to a range of discrete spatial-frequencies, with the assumption being that it will reflect sensitivity to those same spatial-frequencies when they are contained within an image. Sensitivity is the inverse of the threshold contrast, which is the contrast required for the cell to fire at a specified firing rate or, in a behavioral study, to be able to just detect the stimulus.

The unit of measure for spatial frequency is how many sinewave cycles there are within a degree of visual angle (i.e., cycles per degree, or c/deg). The reason for using visual angle, as opposed to a fixed linear distance, like cm, is that these spatial sizes refer to image sizes on the retina, which in turn depends on the size of the object *and* the distance to it. Think about this in relation to your ability to resolve a letter. It will get harder if the letter gets smaller and/or moves farther away, since both of these result in a smaller image on the retina. Thus, we need a measure that takes into account both the size of the object and how far away it is. Visual angle does this by taking the trigonometric tangent function of the ratio of the size of the object and the distance to it. This means that for a given sinewave image, its spatial-frequency content will get higher as it moves farther away (i.e., a higher number of cycles per degree).

**Fig. 2:**
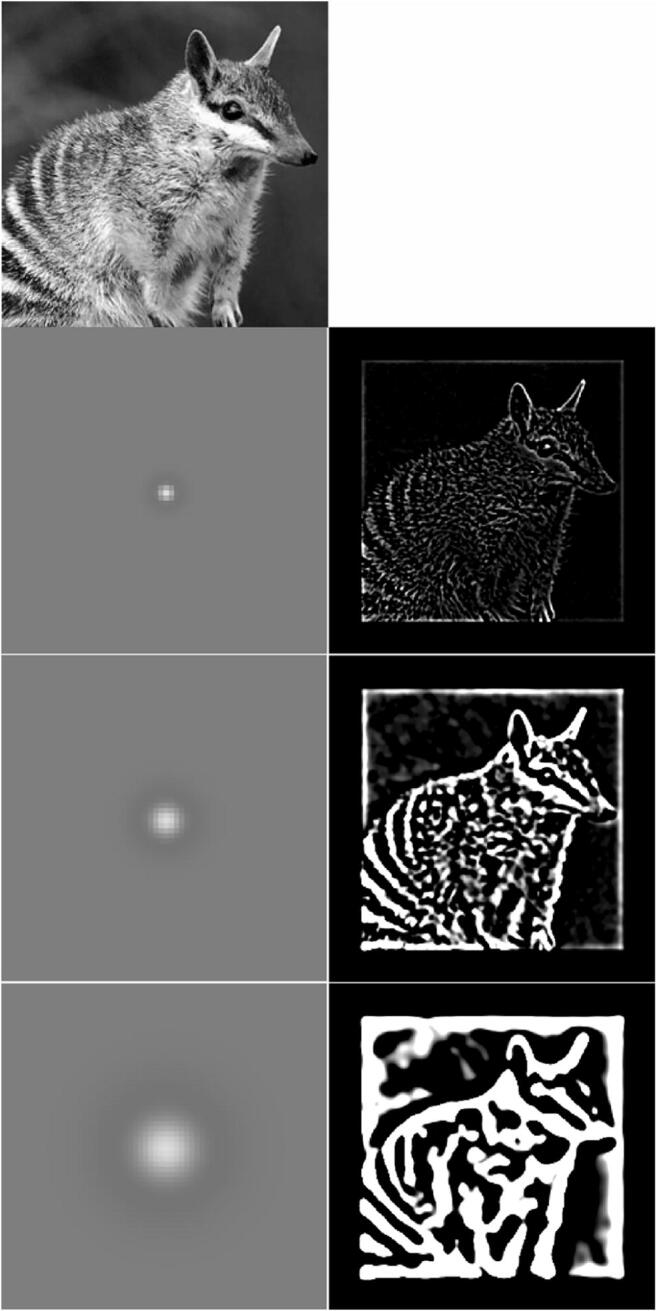
Spatial frequency demonstration. The top figure is a photograph of a numbat (an Australian marsupial). The three lower images are the same image, but they are filtered so that they only contain (from top to bottom) high, intermediate, or low spatial frequencies. This filtering was achieved by convolving the top image with the spatial filter shown on the left: a circular difference of Gaussians where bright regions show excitation and darker regions inhibition. The images on the right are normalized to use the full intensity range but they still show that high frequencies carry information about fine-scale edges in preference to information about luminance changes in larger regions, while lower spatial frequencies do the opposite

High spatial frequencies mean many cycles per degree (and a short wavelength), while low frequencies mean few cycles (and a long wavelength). Hence, high spatial frequencies convey information about fine spatial detail, while low frequencies can only convey coarse detail. For example, when looking at an animal, such as a numbat, the details of the fine fur structure and of the face are conveyed by the high spatial frequencies, whereas the overall gist of the image, like the overall shape and the location of the features on the face, are conveyed by the low spatial frequencies. While the terms “low” and “high” spatial frequency are used, it is important to remember that spatial frequency occurs on a continuum, and these are labels attached to the extreme ends of it, but there are no hard and fast category boundaries (see Fig. 2).

Thus, determining the sensitivity of visual cells to luminance sinewaves with different spatial frequencies provides a direct measure of how sensitive they are to these different aspects of the visual scene. An additional benefit of using a spatial-frequency approach is that visual cells, especially simple cells in area V1, appear to be selectively tuned to spatial frequency, with the preferred spatial frequency of cells varying with their receptive-field size (De Valois, Albrecht, & Thorell, 1982; Field & Tolhurst, 1986; Kulikowski & Bishop, 1981a, 1981b). That is, cells in the early part of the visual system, at least in part, break the visual scene down into different spatial-frequency ranges. This has a superficial similarity to Fourier analysis, but is better thought of as providing an image analysis at different spatial scales at each point on the image. Subsequent processing pools these local analyses into larger perceptual units.

**Fig. 3:**
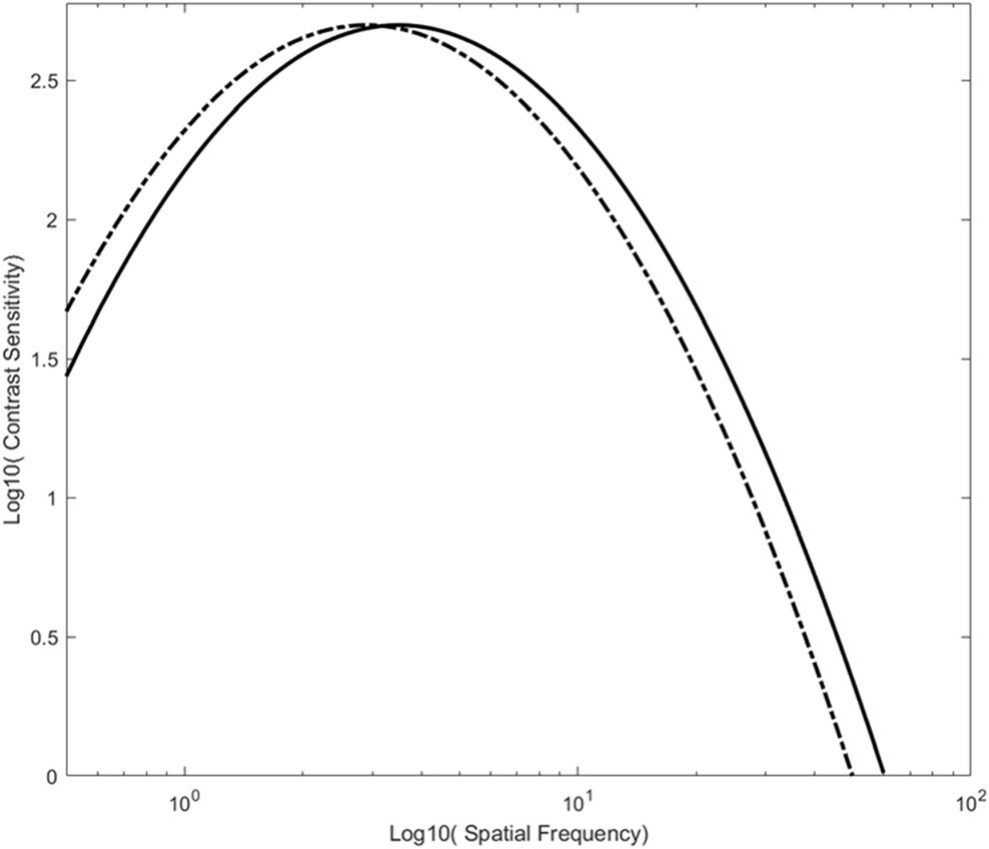
Demonstration of differences in the upper and lower frequency cutoffs of two systems: System A, depicted by the solid line, and System B, depicted by the dashed line. While there is substantial overlap in the spatial frequencies that the two systems are sensitive to (the region in the middle that is encompassed by both tuning curves), System A is tuned to higher frequencies than System B (that is, it has a higher, upper-frequency cut off), and System B is tuned to lower frequencies (that is, it has a lower lower-frequency cutoff). This means that there is a range of high frequencies that only System A is sensitive to and a range of low frequencies that only System B is sensitive to

Studies that have investigated the spatial-frequency tuning of M and P cells have consistently found three things. The first is that all M cells are not tuned to the same spatial frequencies, and not all P cells to the same spatial frequencies. That is, at the population level, both systems are tuned to a range of spatial frequencies, with individual cells being tuned to different parts of that range. The second is that there is substantial overlap in the population tuning properties of M and P cells. That is, the M and P cells do not exclusively process different parts of the spatial-frequency spectrum. However, given that, the third finding is that there are consistent differences in their spatial-frequency tuning, with M cells being biased toward low spatial frequencies and P cells to high spatial frequencies (see below). Given these relative tuning properties, in relation to the fundamental issue of whether it is possible to selectively drive them, the real question becomes, do a subgroup of M cells uniquely process the lowest and a subgroup of P cells the highest spatial frequencies? This can be addressed by determining the lower and upper frequency cutoffs of the cells (i.e., lowest and highest frequencies, respectively, that M and P cells are sensitive to; see Fig. 3). For there to be unique parts of the spatial-frequency range that M and P cells process, cells that have the lowest lower-frequency cutoff would need to be M cells, and cells with the highest upper-frequency cutoff would need to be P cells.

The clearest observed difference is in the higher-frequency cutoff of the cells. It has been consistently shown that the population of P cells contains cells that are tuned to higher spatial frequencies than M cells are, though, since these differences can be small, how that is reported in the various studies differs. Some authors highlight that difference, while others focus on reporting the similarities in their tuning rather than the differences that they had found (Blakemore & Vital-Durand, 1986; Derrington & Lennie, 1984; Kaplan & Shapley, 1982; Levitt, Schumer, Sherman, Spear, & Movshon, 2001). For example, Kaplan and Shapley (1982) highlight the greater high-spatial-frequency sensitivity of P cells. They quantified spatial resolution in terms of the highest spatial frequency at which the response to a drifting sinewave grating (80% luminance contrast, drifting at 4–8 Hz; i.e., cycles per second) disappeared. At matched eccentricities, they found averages of 8.0 c/deg for the 59 P cells, 5.7 c/deg for the 20 sustained (X type) M cells, and 2.5 c/deg for the seven transient (Y type) M cells. On the other hand, Levitt et al. (2001) state that they found “little difference between magno- and parvocellular neurons . . . with respect to these [spatial] variables” (p. 2117). However, they also state that, when looking at the central 5 degrees of the visual field, they found a small, though statistically significant difference between the M and P cells, with the P cells being sensitive to higher spatial frequencies. Additionally, and consistent with this finding, they also found that, at any given eccentricity, the cells that had the smallest receptive fields were the P cells. Note that the spatial frequency that a cell is tuned to is linked to the size of its receptive field: Higher spatial frequencies are processed by cells with smaller receptive field sizes (Sceniak, Hawken, & Shapley, 2002). Hence, the strong finding from all these studies is that while there is substantial overlap in the spatial-frequency tuning properties of the M and P cells, the responses of the population of P cells includes tuning to higher spatial frequencies than the M cells, although the difference is often small.

The studies cited above that found P cells are, on average, tuned to higher spatial frequencies, and they also found that some M cells are tuned to lower spatial frequencies than P cells. For example, Derrington and Lennie (1984) found that M cells have a peak response at lower spatial frequencies than P cells. However, as stated above, the crucial question here is whether M cells have a lower lower-frequency cutoff than P cells. That is, are the lowest spatial frequencies processed exclusively by the M cells in the same way that the highest spatial frequencies are processed exclusively by the P cells? There is no strong evidence from the electrophysiological studies that M cells exclusively process low spatial frequencies. As we will see later, evidence from the lesion studies is a bit stronger (see section Lesion Studies), and the strongest evidence actually comes from human psychophysical studies (see section Pulsed and Steady Pedestals).

However, in relation to this issue, especially as it relates to the human visual system, there are three important issues to consider. The first is the larger size of dendritic fields of parasol (M) cells but not midget (P) cells in humans, compared with monkeys (Dacey & Petersen, 1992). The second is the differences in the sampling densities of the M and P cells, and the third is the actual spatial-frequency bandwidth of the stimuli used to measure their sensitivity.

### Relative size of dendritic fields in humans and monkeys

When using nonhuman-primate electrophysiological studies as a guide for predicting the relative spatial-frequency tuning of human M and P cells, it is important to consider the findings of the study by Dacey and Petersen (1992). In a retinal-staining study on human eyes (obtained post mortem), they found that the dendritic field size of parasol cells (that project to the M cells in the dLGN) are almost twice as big as those in macaques in the central 5 degrees of the retina. Based on this difference, they predicted that human parasol, and hence M cells, should have lower spatial-frequency tuning than M cells in the macaque. In contrast, they found no differences in the dendritic field sizes of the midget (P) cells. This increases the likelihood that the lowest spatial frequencies that humans are sensitive to are processed exclusively by the M cells.

### Sampling densities of M and P cells

The spatial-frequency tuning and resolution of the two systems is not just dependent on the frequency tuning of the individual cells, but also the relative sampling densities of the two systems. This is because images of objects tend to be much larger than the receptive-field size of individual cells, and so the visual system needs to pool across many cells to process those images (Lalor & Foxe, 2010). The height and width of objects in our visual world are typically several degrees of visual angle in size. You can test this by holding your thumb up at arm’s length, which produces an image size of around 2 degrees in width (O’Shea, 1991) and comparing it to the size of images cast by objects around you. However, the receptive field sizes of M and P cells are very small. For example, in the central 5 degrees of the macaque retina, the average receptive-field diameters of M and P cells are 0.089^o^ and 0.069^o^, respectively (Levitt et al., 2001). This means that typical images need to be processed, or sampled, by many dLGN cells and the number of cells that cover a given area across the visual scene, (i.e., the sampling density) limits the spatial resolution of the system. In order for the visual system to resolve a given spatial frequency, it needs to sample it at twice that frequency. This is known as the Nyquist limit, and it means that the greater the sampling rate, the greater the spatial-frequency resolution of a system (Bracewell, 1978; Williams, 1985). The issue of the sampling rate first occurs at the retinal level, with respect to the sampling rate of the cones and rods. As stated above, about 80% of dLGN cells are P and 10% are M. This means that there are substantially more P cells than M cells in the dLGN, and there are similar differences in the relative number of midget and parasol retinal-ganglion cells that feed into these cells (Nassi & Callaway, 2009). Thus, the sampling density of the P system is far greater than that of the M, and so will be another factor (in addition to the spatial-frequency tuning of the individual cells) that results in high spatial-frequencies being selectively processed by the P system for spatially extended stimuli—that is, those greater than about 0.069^o^ (i.e., about 4 minutes of arc; Kaplan, 2004; Lalor & Foxe, 2010; Merigan & Katz, 1990).

### Spatial-frequency bandwidth of stimuli

Finally, in reviewing the evidence for the spatial-frequency tuning of cells and linking it to the performance of the pathways composed of many cells, it is important to consider the spatial-frequency bandwidth of the stimuli used in those studies. Specifically, when trying to determine whether particular cells are tuned to a specific, narrow frequency range, the stimulus used must also be narrowband—that is, it must contain energy only within that frequency range. Consider, for example, trying to determine whether various cells are tuned only to low frequencies (e.g., from 1 to 2 c/deg). If the stimulus used is narrowband (e.g., a spatially extended, sinusoidal grating, and so only contains frequencies within that range), then a cell that was sensitive to those low frequencies would respond to it while another cell, tuned to higher frequencies (e.g., 3 to 4 c/deg) would not. This means that the interpretation of the data from these stimuli would be valid. However, what if the stimulus contained most of its energy at those low frequencies (1 to 2 c/deg), but also contained energy at higher frequencies (3 to 4 c/deg)—that is, it was a more broadband stimulus? Such a stimulus would drive cells tuned to low frequencies and also those tuned to high frequencies. This would be a particular problem if you thought that your stimulus only contained low frequencies and the results were interpreted accordingly (Campbell & Robson, 1968; De Valois, De Valois, & Yund, 1979). The only stimulus that contains just the intended frequency (e.g., 1 c/deg) is a perfectly formed, continuous sinewave of infinite extent. Any distortion to that sinewave profile or spatial truncation of it (so that it only extends over a restricted region of space) increases the range of frequencies in it (i.e., it makes it more broadband; Bracewell, 1978). We will discuss these issues in more detail in the section Spatial Frequency and Stimulus Bandwidth.

The researchers who have conducted these electrophysiological studies have typically conducted their experiments to minimize the effects of these issues—for example, by using slides of sinewaves to minimize image distortions (Hicks, Lee, & Vidyasagar, 1983) and by ensuring that the projected image of the sinewave extends beyond the spatial extent of the cell’s receptive field (Badcock, 1990; Blakemore & Vital-Durand, 1986). However, distortions in the sinewave profile and variations in the spatial extent of the stimulus invariably occur in studies resulting in a broadening of the bandwidth of the stimuli (Badcock, 1990). This issue of relatively broad stimulus bandwidths is a potential reason for the failure to find selective processing of low spatial frequencies by M cells. This is because (as discussed above) if there is this M cell selectivity at low spatial frequencies, it occurs over a narrower frequency range than the P selectively at high spatial frequencies.

The electrophysiological evidence reviewed so far strongly supports the idea that very high spatial frequencies are selectively processed by P cells, while there is no strong evidence that static, low frequencies are selectively processed by M cells. We will now look at the relative tuning of the M and P cells to temporal frequency and contrast, and also consider how these stimulus parameters impact spatial-frequency tuning.

## Temporal-tuning properties

Temporal information refers to changes in luminance or color at a particular spatial location over time. In parallel with how sensitivity to spatial information is quantified, temporal sensitivity is also usually expressed in terms of sensitivity to different temporal frequencies—that is, sensitivity to sinusoidal variations in the local mean-luminance (or color) levels over time, with the unit of measure being hertz (Hz; i.e., cycles per second). Results from some studies indicate clear differences in the population tuning of M and P cells to temporal frequency. At a population level, M cells are tuned to higher temporal frequencies than P cells are (Derrington & Lennie, 1984; Levitt et al., 2001). For example, Levitt et al. (2001) found that the highest temporal frequency that would still drive macaque cells to half their maximum response was 31.6 Hz for M cells and 21.9 Hz for P cells.

Other differences in temporal properties relate to the nature and the latency of their response. M cells give a transient response, in that they briefly respond to either the onset and/or offset of the stimulus, while P cells give a more sustained response over the entire duration of the stimulus (Kaplan, 2004; Yeh et al., 1995). M cells also have shorter response latencies (Kaplan & Shapley, 1982; Levitt et al., 2001; Schiller & Malpeli, 1978). For example, Levitt et al. (2001) found that for cells in the central 5 degrees, M cells had a latency of 28 ms and P cells 68 ms. That is, M cells fire about 40 ms more quickly than P cells, though others have found latency differentials of only 10 ms (Maunsell et al., 1999). Also, the larger axons of the M cells typically result in faster axon conduction speeds compared with P cells. Both of these factors mean that M cells can transmit information to the primary visual cortex more rapidly than P cells, with one estimate being M cells can get information to V1 10-ms quicker than P cells (Maunsell & Gibson, 1992).

In summary, M and P cell populations have response maxima in different regions of the spatiotemporal frequency spectrum and thus their contrast responses will contribute to the overall system’s response to a varying extent, depending how the stimulus properties match these differences. These interactions have been explored more fully in lesion studies and will be discussed below.

### Color tuning

Another important difference between M and P cells relates to their sensitivity to color information. Indeed, an early name for them was based on differences in their relative tuning to the wavelength of light (which our perception of color is based on). Note, here we are referring to the actual wavelength of the light (sinewave modulations in electromagnetic radiation) and not the sinewave variation in luminance across the image. A retinal or dLGN center-surround cell becomes tuned to a particular stimulus dimension by having its center and surround regions of its receptive field be responsive to different values along that dimension. As discussed above, these cells are responsive to luminance differences because their center and surrounds are differentially sensitive to luminance, excitatory versus inhibitory. The same occurs for sensitivity to color differences. The center and surround of M cells receive equal inputs originating in both long and middle wavelength-sensitive cone receptors, and so they have no differential sensitivity to color. Both regions are sensitive to a broad range of wavelengths, and hence they were labeled broadband-sensitive cells. P cells, on the other hand, have differential inputs to their center and surround. For example, long wavelength sensitive cones may (predominantly) feed into the center, while middle wavelength sensitive cones may (predominantly) feed into the surround. Hence, each region is preferentially sensitive to a different range of wavelengths—the cell is sensitive to color differences between the center and surround, and so they were called color-opponent cells. All P cells seem to be sensitive to color differences while M cells are not, though some M cells have been reported to have an inhibitory red surround, and hence their response can be inhibited by diffuse red light (De Valois, Abramov, & Jacobs, 1966; Wiesel & Hubel, 1966). This means that P cells mediate our perception of color, while M cells do not. When considering these issues, it is important to keep in mind that an image of an object is typically defined by differences in both its color and luminance compared with its background. For example, when looking at a red bar on a green background, there is the obvious color difference, but there is essentially always a luminance difference as well. The red bar might have a greater luminance (be brighter) than the background. M cells are sensitive to just the luminance difference, while P cells are sensitive to both the color and the luminance differences.

Note that some studies attempt to create stimuli that contain only color information to constrain the signal processing to P cells. In the above example, that would consist of matching the luminance of the red bar to that of the green background, creating an isoluminant stimulus. The logic of these studies is elegant. Given the insensitivity of the M cells to color information, if a particular perceptual attribute, like motion, could be perceived with isoluminant stimuli, then that would be powerful support for a role of P cells in mediating the perception of that attribute (Cavanagh & Anstis, 1991; Cavanagh & Favreau, 1985; Derrington & Badcock, 1985; Livingstone & Hubel, 1987). The technique called heterochromatic flicker photometry is used to match the luminance of different colors. This entails alternating between the two colors at a high rate (e.g., 15 Hz). When this is done, the percept is one of a single, constant color (which is the additive sum of the two colors), and if there are luminance differences between the two colors, a flicker will be perceived in the luminance of that single color. The luminance of one of the colors is then adjusted to remove, or at least minimize, that luminance flicker (Wyszechi & Stiles, 1982). This logic of this technique is based on the perception of color being mediated by the P system, which is not very sensitive to high (15 Hz) temporal frequencies (Lee, Pokorny, Smith, Martin, & Valberg, 1990), while luminance perception involves both M and P cells. Thus at 15 Hz, the P cells cannot resolve the alternating colors, and so a combined color is perceived, while the M cells can resolve the luminances of the two colors, and so any difference in them is perceived as a luminance flicker (Cropper & Badcock, 1994; Wyszechi & Stiles, 1982). Unfortunately, as we will discuss below, it is not possible to construct stimuli that are isoluminant at every location, especially when large, moving stimuli are used (Cropper & Derrington, 1994; Derrington & Badcock, 1985; Edwards & Badcock, 1996).

### Interim summary

Based on these electrophysiological studies, a strong case can be made for P cells giving the visual system sensitivity to high spatial frequencies combined with low temporal frequencies and color information, and M cells mediating sensitivity at high temporal frequencies combined with low spatial frequencies at low luminance contrast. Further, a weaker case can be made for P cells giving the visual system discrimination ability to contrasts above 20% (i.e., the response of P cells not saturating to increasing contrast above 20% while M cells do, though again, this is debated; Kaplan, 2012; Kaplan & Shapley, 1986). A strong case for M cells mediating sensitivity at low spatial frequencies cannot be made, though this conclusion will be qualified when we have considered the effect of temporal frequency on this relative selectivity.

## Lesion studies

As stated above, it can be difficult to infer the sensitivity ranges of the M and P systems based on the tuning properties of a very limited sample of cells. Lesion studies, on the other hand, promise to indicate what the entire population of cells in a given pathway processes. The logic of this approach is essentially to damage (“lesion”) an entire pathway, and examine how visual functioning is impaired as a result of removing the information processing contributed by that pathway. These studies typically use excitotoxins like ibotenic acid, which damage cell bodies, but not the white matter, or acrylamide, which selectively lesions the P cells at either the retinal or dLGN level (Merigan & Maunsell, 1993) and are conducted in nonhuman primates.

The strong conclusions from the electrophysiological studies are supported by the results of lesions studies. Sensitivity to both luminance information at high spatial frequencies and to color information are greatly impaired, if not entirely lost, by lesions to the P cells, while lesions to M cells have no impact (Merigan & Eskin, 1986; Merigan, Katz, et al., 1991b; Merigan & Maunsell, 1993; Schiller et al., 1990b). For example, P-cell lesions resulted in a 3–4-fold reduction in spatial acuity, while M-cell lesions had no effect on spatial acuity (Merigan & Katz, 1990). Spatial acuity was determined by the highest frequency luminance sinewave that could be resolved, when modulated at 55% contrast. Similarly, color discrimination of targets was “devastated” by P lesions, but not affected by M lesions (Schiller et al., 1990b). Note that conditions were run in which the stimuli were made isoluminant, or as close to it as possible, but more importantly, given the issues in actually generating an isoluminant stimulus (Edwards & Badcock, 1996), the different colors were presented at a number of different luminance-contrast combinations so that luminance contrast was not a reliable cue to the different colors. M-cell lesions, on the other hand, had a significant impact on temporal acuity, but P-cell lesions did not. For example, temporal acuity, as measured by critical-flicker-fusion thresholds (i.e., the highest flicker rate of a uniform luminance screen that can be detected) was severely reduced following M-cell lesions, but was not affected by P-cell lesions (Schiller et al., 1990b). These lesion studies thus provide further evidence that color perception and spatial acuity (sensitivity to high spatial frequencies) are mediated by P cells, with no contribution from M cells at detection threshold, while temporal acuity (sensitivity to high temporal frequencies) is mediated by M cells, with no contribution from P cells at threshold levels.

Consistent with the sensitivity of M cells to high temporal frequency, M cells seem to play a major role in motion processing, especially for low-contrast and high-speed stimuli. While P lesions had minimal impact on motion direction discrimination, M lesions had a significant impact, with the magnitude of the effect varying with the luminance contrast, spatial frequency and speed of the stimuli, consistent with the above findings. When the contrast of the stimulus was below about 30% contrast, detection and discrimination ability were abolished, and even at the highest contrasts, performance was still significantly impaired. Similarly, performance was most impaired at higher speeds (above 1 deg/s) (Merigan, Byrne, et al., 1991a; Schiller et al., 1990b). Note that for a grating at a given spatial frequency, increasing the speed of that grating increases the number of bars that move past a given point in a second, so it also increases its temporal frequency.

Turning now to the question of whether M cells selectively process low spatial frequencies, the evidence for it is mixed. However, as with the electrophysiological studies, there are potential issues with the spatial-frequency bandwidth of the stimuli. For example, Schiller and colleagues (Schiller et al., 1990b; Schiller, Logothetis, & Charles, 1990a) argue that both M and P cells process low spatial frequencies, but the stimuli used to draw those conclusions were square-wave gratings presented with hard edges. This is problematic for two reasons. The first is that, as discussed previously, having hard edges on the stimulus will increase the spatial-frequency bandwidth of that stimulus (see Fig. 5). Additionally, instead of using sinewave gratings, they used checker-board patterns (i.e., square-wave patterns). The Fourier spectrum of a square wave consists of a sinewave at the frequency of the square wave (called the fundamental frequency, and in their study the lowest square-wave frequency they used was 1.54 c/deg), but also sinewaves at the odd harmonics—that is, odd multiples of the fundamental (so 3, 5, 7 etc.) times the fundamental (so 4.62, 7.70 and 10.78c/deg) with the contrast of those harmonics decreasing with increasing frequency. The contrast of each harmonic is equal to contrast of the fundamental (i.e., the sinewave that has the same wavelength of the square wave) multiplied by 1 divided the harmonic number (e.g., the third harmonic has a contrast one-third that of the fundamental frequency, the fifth is one-fifth). Both of these factors (hard edges and square-wave gratings) mean that the spatial-frequency bandwidth of their stimuli would have been broad, so the lowest frequency that they tested (nominally 1.54 c/deg) would have actually extended to much higher frequencies so that they would have driven the P cells. This could occur because cells tuned to frequencies near the peak of the contrast sensitivity function will have higher sensitivity and could potentially detect on of the higher harmonics of the square wave grating to determine the contrast threshold. Thus, even if M cells do selectively process low spatial frequencies, these studies would not have been able to determine it.

The studies by Merigan and colleagues (Merigan & Eskin, 1986; Merigan, Katz, et al., 1991b ; Merigan & Maunsell, 1990), at least in part, address issues of stimulus bandwidth. While some of their studies used sinewaves that had abrupt (i.e., hard) edges, others used Gabor patterns—that is, a sinewave grating presented in Gaussian spatial window. This stimulus has a narrower bandwidth than one with hard edges (see section Spatial Frequency and Stimulus Bandwidth, below).

However, even when Gabor stimuli were used, there was still no strong evidence that M cells process the lowest spatial frequencies when the stimuli were static (i.e., when the stimuli essentially had a temporal frequency of zero). Note that the stimuli were presented in a raised-cosine temporal window (i.e., the contrast of the stimuli was gradually increased from zero) to reduce the temporal frequency associated with briefly presenting the stimuli. This is the temporal equivalent of the Gabor’s Gaussian spatial window. This situation changed when the temporal frequency of the stimulus was increased. Then, there was clear evidence that low spatial frequencies are processed by M cells. Thus, consistent with the findings outlined above for relative sensitivities to temporal frequency, results indicated that M cells are preferentially sensitive to the combination of low-spatial and high-temporal frequencies and P cells are preferentially sensitive to the combination of high-spatial and low-temporal frequencies. For example, following P cell lesions, sensitivity to a 0.7 c/deg stimulus was impaired at the lowest temporal frequencies, 0.5 and 1Hz (indicating P mediation), but not for higher frequencies (M mediation). For the for 3.4c/deg stimulus, performance was impaired for frequencies up to just under 10 Hz—that is, impairment over a broader frequency range, indicating the greater role of P cells in processing the higher spatial frequency. Consistent with this, a 15-c/deg stimulus was impaired for all temporal frequencies (Merigan & Eskin, 1986).

Lowering the contrast of the stimuli made it more likely that, at a given spatial and temporal frequency, performance would be reliant on M cells (Merigan, Katz, et al., 1991b; Merigan & Maunsell, 1990). Thus, with respect to luminance-contrast sensitivity, the results map onto the relative spatial and temporal sensitivities of the M and P cells. For example, M-cell lesions dramatically impacted contrast sensitivity for stimuli at high-temporal and low-spatial frequencies, but did not affect sensitivities to stimuli at high spatial frequencies (Merigan, Byrne, et al., 1991a). Note that a uniform screen, as used in critical-flicker-fusion thresholds, is defined by very low spatial frequencies, essentially 0 c/deg—this interplay between temporal and spatial frequencies has also been demonstrated in masking studies in humans (Badcock & Sevdalis, 1987; Yang, Qi, & Makous, 1995). These results are consistent with high spatial frequencies being selectively processed by P cells. Similarly, sensitivity to low temporal frequencies is not affected by M lesions, given the sensitivity of P cells to low temporal frequencies (Merigan & Maunsell, 1993). In terms of contrast (i.e., brightness) discrimination, M lesions had no effect on performance, while P lesions resulted in small (one animal) or no (second animal) impairment when the target was higher contrast to the other stimuli, but P lesions resulted in a major impairment when the target was lower contrast than the other stimuli (Schiller et al., 1990b).

Finally, the loss of sensitivity to moving stimuli following M or P lesions maps onto the relative spatial and temporal frequency sensitivity of the cells. Thus, following M lesions, direction discrimination of a 1-c/deg Gabor was not impaired when it was moving at 1 degree/s (i.e., 1 Hz), but it was at higher speeds (and hence higher temporal frequencies—temporal frequency is equal to spatial frequency multiplied by speed). This led Merigan and colleagues to conclude that sensitivity to motion was not specifically impaired by M lesions, but rather that the impairment in motion sensitivity resulted from the reduced detectability of the stimuli when they were defined by the appropriate combination of temporal (high) and spatial (low) frequencies (Merigan, Byrne, et al., 1991a), which is consistent with human psychophysical evidence for separate pathways for static, low, and high speed motion (Edwards, Badcock, & Smith, 1998; Khuu & Badcock, 2002).

## Unique roles of the M and P pathways

There are strong consistencies between the results of both the electrophysiological and lesion studies regarding the relative tuning and functional specializations of the M and P pathways—namely, that while there are significant similarities between their tuning, there are also significant differences. The clearest findings relate to spatial frequency, temporal frequency, and color tuning. P cells process high spatial frequencies and color information, while M cells do not. M cells, on the other hand, process high temporal frequencies and low luminance contrast, while P cells do not. Though it is possible that, while individual P cells are not sensitive to low luminance contrasts, the P system as a whole is, through the pooling of the responses of the large number of P cells (recall that 80% of dLGN cells are P cells; Kaplan, 2012). This idea of contrast sensitivity of cells further along the visual system being enhanced due to neural convergence is supported by the finding that the contrast-response gain of cells in macaques increases as the measurements move from the dLGN to V1 to MT/V5 (Sclar, Maunsell, & Lennie, 1990). In relation to contrast, there is not strong support for the idea that P cells mediate contrast discrimination at high (above 40%) luminance contrast. Finally, the extent to which M cells selectively mediate sensitivity to low spatial frequencies is debatable. At best, there would appear to be less separation in the tuning properties of M and P cells at low, compared with high, spatial-frequencies, though, as noted above, the spatial tuning bandwidth of the stimuli used to test their relative tuning may have limited the findings. Additionally, the temporal frequency of the stimuli plays an important role, with selective M cells processing becoming apparent when the temporal frequency is raised above 1 Hz.

This means that the initial claims of the degree of specialization of the pathways were excessive, like, for example, claiming motion perception was mediated *purely* by M cells and form perception by P cells (M. Livingstone & Hubel, 1988; Ungerleider & Mishkin, 1982). However, there are significant differences between the sensitivities of the M and P cell populations, and, hence, specialization of function. Schiller and colleagues’ conceptualization of the differences in terms of the P system pushing the sensitivity of the visual system toward high spatial frequencies and color selectively, and the M system toward higher temporal frequencies, seems apt (Schiller et al., 1990a, 1990b). Their differences with respect to color processing is also reflected in the alternative name for the M and P cells—broad-band and color-opponent, respectively. As stated previously, broad-band here refers to their tuning to wavelength in the electromagnetic spectrum—that is, hue (color) and not to luminance-defined spatial frequency. Note that the there are differences in the P-dominated blob–thin-stripes and interblob–interblob streams in relation to the processing of color information. The main difference in their properties is in relation to orientation tuning. Cells in the blobs are not tuned to orientation while those in the interblob regions are (H. D. Lu & Roe, 2008; Roe & Ts'o, 1999). Additionally, the thin stripes in V2 are spatially arranged such that there is a systematic mapping of cells according to their preferred color tuning, which further indicates that this area is involved in the processing of perceived color (Xiao et al., 2003). This has led to the idea that the blob–thin-stripe pathway is involved with processing the surface properties of objects, both color and brightness (luminance), while the interblob–interstripe pathway processes the contour (shape) information of objects based on color and luminance differences (H. D. Lu & Roe, 2008; Van Essen & Gallant, 1994). Again, though, in relation to functional segmentation of M and P pathways, keep in mind that P cells provide dominate input to the blob–thin-stripe and interblob–interstripe pathways, and M cells to the thick-stripe pathway, they do not do so exclusively, and that there is a degree of interaction between these pathways (DeYoe & Van Essen, 1988). Based on the electrophysiological studies, other clear differences are that M cells can give either transient or sustained responses, P cells only give sustained responses, (although the salient part of the response may be at the beginning with a very strong stimulus; Samonds & Bonds, 2004) and that the response latency of M cells is shorter than that of P cells (Kaplan, 2004; Yeh et al., 1995).

## Tailoring visual stimuli to selectively drive the M and P systems

The clear conclusion from the above studies is that, while there is a great deal of overlap in the tuning properties of the M and P systems, there are also specific differences. It is not the case that the processing of specific visual attributes like motion or form can be allocated exclusively to particular pathways (Cropper, 2006; M. Livingstone & Hubel, 1988; Ungerleider & Mishkin, 1982). Similarly, viewing a complex task like reading as being the sole domain of one of these pathways is also incorrect, given the degree of interaction between them (Nassi & Callaway, 2009). However, given the established differences in their tuning properties, it is also incorrect to argue that it is *not* possible to selectively drive them. That said, given the overlap in their tuning properties, great care needs to be taken to selectively drive them. We will now outline the various ways that visual stimuli can be tailored to selectively, or at least preferentially, drive them.

### Temporal and spatial acuity

The clearest differences between the M and P cells lies in the highest spatial and temporal frequencies that they are sensitive to. M cells are sensitive to high temporal frequencies, while P cells are not, and P cells are sensitive to the highest resolvable spatial frequencies, while M cells are not. Thus, one of the easiest ways to selectively tap them is to use temporal and spatial acuity tasks—that is, tasks that tap the greatest temporal and spatial resolution of the visual system. However, as the lesion studies clearly showed, there is an interaction between these two parameters such that M and P cells are sensitive to particular combinations of temporal and spatial frequencies (Merigan & Eskin, 1986; Merigan & Katz, 1990; Schiller et al., 1990b). This means that, for example, merely using a temporal-frequency task will not necessarily tap the M cells. The spatial-frequency content of the stimulus being used is also important. Specifically, it must be one whose spatial frequency content is centered on low frequencies, rather than high. If the stimulus is defined by high spatial frequencies, then P cells will mediate the measured temporal acuity. Similarly, if a spatial-acuity stimulus is defined by high temporal frequencies, then M cells will mediate the measured spatial acuity (Merigan & Eskin, 1986).

These considerations are particularly important given that most studies that use spatial-acuity and temporal-acuity tasks to selectively tap the M and P pathways do not establish threshold performance (i.e., the highest spatial and temporal frequencies that can be detected). Instead, they determine how performance on acuity tasks (using stimuli whose spatial and temporal properties are fixed) vary when the process of interest (e.g., spatial attention) is manipulated. For example, spatial-acuity tasks typically involve detecting the presence/absence of a spatial gap in a circle or discriminating a letter (e.g., *E* vs. *F*), and temporal-acuity tasks the presence/absence of a temporal gap in the presentation of a stimulus (i.e., was the stimulus presented continuously or did it pulse off briefly in the middle of the presentation period; Badcock & Lovegrove, 1981; Bocanegra & Zeelenberg, 2011; Bush & Vecera, 2014; Coltheart, 1980; Goodhew et al., 2016; Gozli et al., 2012; Yeshurun & Levy, 2003).

Thus, when conducting these types of experiments, *all* of the stimulus parameters over which the M and P cells differ in their respective tuning need to be considered when selecting the actual stimuli to be used. For example, if a spatial-acuity stimulus is being used to tap the P cells, then all of the stimulus parameters need to be optimized in order to ensure that the P cells are mediating performance. This means that the stimulus should not contain just low spatial-frequencies and/or have a low luminance contrast, because that would decrease the sensitivity of the P cells to such a stimulus. Similarly, the stimulus should not be presented for only a very brief duration. Instead, the stimulus should be defined by high spatial frequencies and a high contrast and be presented for a relatively long duration. Note that M cells are also strongly driven by high luminance-contrast stimuli (it is P cells that are not very responsive at low contrasts), but the stimuli need to have the appropriate spatial-frequency and temporal-frequency values. If acuity performance was being mediated by M rather than P cells, then the gap required for the observer to resolve would be expected to be larger.

So, what sort of spatial-acuity stimulus should be used? As stated above, it should have a luminance contrast and stimulus duration that strongly drives P cells, so a contrast of 20% or more (Kaplan, 2012) and a duration of around 100 ms or longer. Note that it is harder to define a critical value for duration required to effectively drive the P system, but psychophysical studies on humans have shown that P-mediated performance improves up to about 100 ms (Pokorny & Smith, 1997). Essentially you do not want to use a very brief duration (e.g., around 25 ms) that will bias the stimulus to driving M, over P, cells (Kaplan & Shapley, 1982). Also, performance on the task needs to depend on resolving high spatial frequencies, so a simple detection task is not ideal, even if the stimulus used is a ‘small’ dot. Such a stimulus is spatially broadband, and so, especially if it is pulsed on using a temporal window with hard edges (i.e., the contrast is not slowly increased using, for example, a raised-cosine temporal window), it will also contain high temporal-frequency information and so will be likely to be detectable by the M cells (Legge, 1978; Leonova, Pokorny, & Smith, 2003; Pope, Edwards, & Schor, 1999; Tolhurst, 1975). An advantage of using a spatial-gap stimulus rather than a standard letter stimulus is that the precise size of the feature that needs to be resolved to do the task is straightforward to quantify. That is, the size of the gap quantifies this information, and hence it is easier to ensure that the task actually requires fine spatial resolution, rather than, for example, discriminating the overall shape of the letter, which may depend on lower-spatial frequencies, and hence tap M cells. This advantage is also true for the tumbling *E* chart, in which only the letter *E* in various orientations is used and the person has to indicate the orientation of the *E*. Here, it is the thickness of the lines of the letter (the stroke width) that defines the critical feature.

Similar issues apply when temporal-acuity stimuli are being used to tap the M cells. Given that M cells are less sensitive to high spatial frequencies, the stimulus used should not be defined by high spatial-frequencies (e.g., a high spatial-frequency grating). Otherwise, the measured temporal acuity will be mediated by P rather than M cells. An example of this spatial frequency dependency is its role in the perception of motion. As was discussed previously, sensitivity to motion, and particularly high-speed motion, is dependent on the involvement of M cells, and motion perception in humans has been shown to be dependent on the presence of low-spatial frequency information. Impairments in motion perception occur when the frequency of a moving grating is increased from 1 to 4 c/deg (Boulton & Baker, 1991; Ramachandran, Ginsburg, & Anstis, 1983; Smith & Edgar, 1990), and it becomes very difficult to discern the motion of gratings near the (P mediated) spatial acuity limit (Badcock & Derrington, 1985). Similarly, the stimulus should not be presented in a long-duration Gaussian or raised-cosine temporal window, otherwise the temporal transients required to drive the M cells will not be present, and again, P cells will be tapped (Pope et al., 1999).

In implementing these types of tasks, it is also preferable to use discrimination rather than detection paradigms. For example, telling the participant that a gap is present and requiring them to indicate its location—for example, the top or the bottom of the circle (discrimination), as opposed to asking them whether there is a gap present or not (detection). Discrimination tasks are a type of forced-choice procedure, and hence remove the subjective criterion of the person from the process. That is, how confident they need to be before they are willing to state that they can detect the stimulus. This removes a potential source of variance in group-based data.

That said, one common reason for using a spatial gap-detection task is to make it similar to a temporal gap-detection task. This task consists of a stimulus that is either continuously presented (no temporal gap) or presented with a brief blank screen in the middle of the presentation (temporal gap). A temporal-order-judgement task is also an effective way to tap the M system. This consists of the rapid presentation of two spatially offset stimuli, and the task is to indicate which stimulus was presented first (Goodhew, Lawrence, & Edwards, 2017; Hein, Rolke, & Ulrich, 2006). Though, note that care should be taken to ensure that the spatial and temporal parameters used do not result in the percept of apparent motion, thus turning the experiment into a discrimination task. While motion should still be processed by the M pathway, if a small spatial offset between the two stimuli is used, then this may result in P cell mediation of the task (due to the fine spatial resolution required).

In relation to the question of what actual values to use for these parameters, while it is possible to be fairly definitive for luminance contrast (below 10% to bias toward M processing and above 20% to strongly drive P cells—though, of course, also M cells, based purely on a consideration of luminance contrast), it is harder to do so for something like critical feature size. To preferentially tap P cells, the feature needs to be defined by high spatial frequencies, but for a gap (as opposed to a Gabor stimulus—see below), what does that really mean? In practice, it is more about being aware of the issues and to use critical features that are about 1 minute of visual angle in size (i.e., around the spatial-acuity limit), and to also explore the effect that varying the critical-feature size has on the pattern of results when pilot testing the experiment. That is, if a qualitatively different effect was observed as stimulus size was increased, it may reflect going from performance being mediated by one type of cell type (e.g., P) to another (e.g., M).

**Fig. 4:**
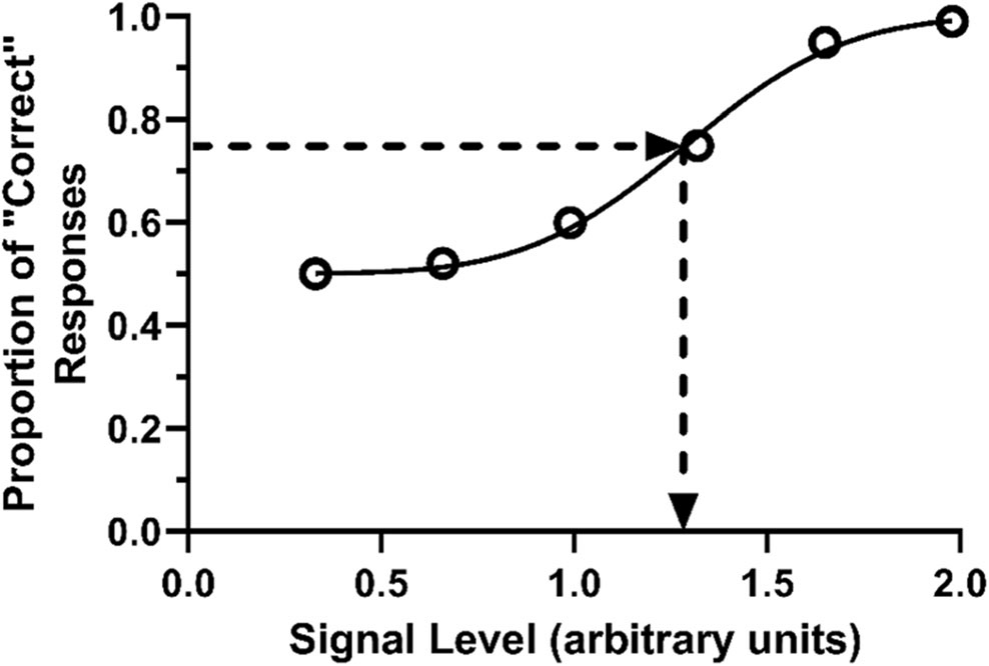
Demonstration of a psychometric curve. Performance (proportion correct responses) is plotted against signal level—that is, stimulus intensity. A two-alternative forced-choice (2AFC) experiment is being depicted, so performance varies from chance level (0.5, i.e., 50%) at subthreshold signal levels to perfect performance (save for the occasional error) at high signal levels. The dotted line indicates the point at which the slope of the curve is the steepest

As stated above, these spatial and temporal acuity studies typically involve determining how performance varies as a function of a cognitive process of interest. In conducting these studies, optimization, and a degree of pilot testing of the specific parameters used is required, not only to maximize the likelihood that they selectively drive the particular system of interest (M or P), but that they produce a performance level that results in the experiment being the most sensitive it can be to the experimental manipulation. For studies that use a forced-choice procedure, the ideal performance level is midpoint between chance level and 100% performance (Goodhew & Edwards, 2019). For a two-alternative forced-choice (2AFC) procedure, that would be the 75% performance level (chance level is 50%). Why this is the case can be explained with reference to the psychometric curve. A psychometric curve is a graph of performance as a function of increasing stimulus intensity (see Fig. 4). Performance goes from chance level at subthreshold intensity levels to 100% performance at substantially supra-threshold levels and the slope of the curve is steepest at the midpoint of the curve (i.e., midway between chance and 100% performance). In other words, the midpoint on the curve is where the smallest change in stimulus intensity results in the greatest change in performance. If the psychological attribute being manipulated in the study (e.g., the size of the spatial region over which attention is allocated) has an effect on the sensitivity of the visual system to high-spatial frequencies (e.g., Lawrence et al., 2020), this can be thought of as being equivalent to keeping the sensitivity of the system constant, and changing the stimulus intensity. Therefore, starting at this performance midpoint will result in the most sensitive measure of the effect of that manipulation. Additionally, the midpoint allows for the detection of enhancements and decrements in performance due to the underlying manipulation.

In summary, using temporal and spatial acuity stimuli are effective ways to selectively tap the M and P cells; however, the entire stimulus parameter space needs to be considered. To tap M cells, one needs to employ a task that requires the resolution of high temporal frequencies, but which are produced by a stimulus that is also defined by low spatial frequencies. Using a stimulus defined by a low luminance contrast will also increase the likelihood of selectively driving the M cells. On the other hand, to tap P cells one needs to employ a task that requires the resolution of high spatial-frequencies, but which are produced by a stimulus that contains high luminance contrast, and low temporal frequencies.

### Spatial frequency and stimulus bandwidth

Another way to selectively tap the M and P cells, which is often suggested, is to use stimuli that are narrowband in the spatial frequency domain so that they contain only low or high spatial frequencies, respectively. However, this approach is more problematic than using temporal-acuity and spatial-acuity tasks for a number of reasons. The first is that, as discussed above, while the selective processing of high spatial frequencies by P cells is well established, the selective processing of low spatial frequencies by M cells is less so.

The second issue is that generating narrowband stimuli is far more difficult than generating temporal and spatial acuity stimuli. There is an intrinsic conflict between making a stimulus that is localized in both the spatial domain (i.e., that is small) and the spatial-frequency domain (i.e., that has a spatial-frequency spectrum that has a narrow bandwidth). As stated above, the stimulus with the narrowest spatial bandwidth is a luminance sinewave that extends to infinity. Its spatial-frequency spectrum consists only of energy at the frequency of the sinewave (i.e., it has a bandwidth of zero). As soon as that stimulus is truncated, its bandwidth is increased. Those additional frequencies represented by that broader bandwidth are the sinewaves (at various spatial phases) that need to be added to the original infinite sinewave in order for the physical stimulus to be transformed into one that only contains luminance modulation within a localized spatial window (Weisstein, 1980).

**Fig. 5:**
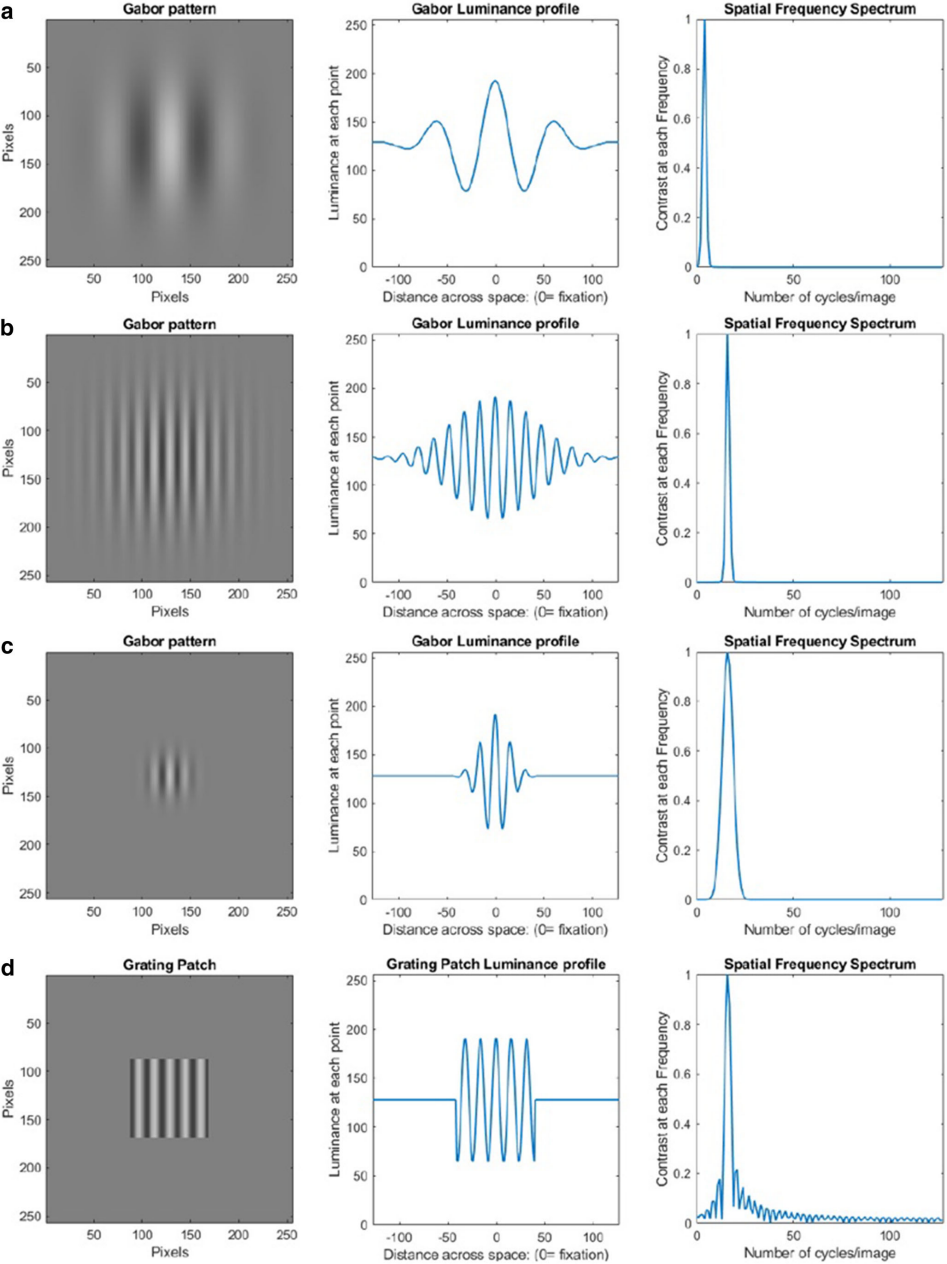
Gabor stimuli (**a–c**) and a sinewave grating in a square envelope (**d**) and their corresponding luminance profiles and their horizontal spatial frequency content. In going from **a** to **b**, the spatial frequency of the Gabor is increased while the size of the Gabor (standard deviation of the Gaussian window) is held constant. The spatial frequency bandwidth stays the same, but it is now centered on the higher frequency. In going from **b** to **c**, the size of the Gabor is decreased while the spatial frequency is held constant. The spatial frequency bandwidth is increased. **d** A sinewave grating that has the same spatial frequency as the Gabors in **b** and **c** but it is presented in a square spatial window (i.e., one with hard edges). This results in a much broader spatial-frequency bandwidth compared with **b** and **c**

The type of spatially localized stimulus that has an optimal combination of localization in both space and spatial frequency is a Gabor, and so it is the most common stimulus used when spatial frequency needs to be controlled. That is, a Gabor stimulus minimizes both the required spatial extent and the spatial bandwidth of the stimulus. Note that the receptive-field profile of simple cells in V1 can also be modeled using Gabor functions, which is taken as further evidence that those cells are tuned to both the spatial location of stimuli and to their spatial-frequency content (Field & Tolhurst, 1986; Kulikowski & Bishop, 1981a, 1981b). A Gabor consists of a sinewave grating whose contrast is multiplied by a Gaussian envelope. That is, it has the highest contrast at the center of the Gaussian, and it gradually reduces to zero (i.e., no luminance modulation, just a mean luminance the same as the background) at the border of the Gaussian. This gradual decrease in the contrast of the sinewave results in a stimulus that has a frequency spectrum that is centered on the frequency of the sinewave, whose bandwidth is also a Gaussian and whose width (standard deviation) is inversely proportional to the standard deviation (width) of the Gaussian of the Gabor. Thus, the larger the Gaussian of the Gabor, the smaller the Gaussian of the tuning bandwidth, and the tighter the tuning bandwidth. This makes logical sense, given that a Gabor with an infinite Gaussian would produce an infinite sinewave, and so have a zero bandwidth. Conversely, the smaller the Gabor the broader its spatial-frequency tuning. For example, if the Gabor has a standard deviation of 2 deg, then it has a spatial-frequency bandwidth of 0.5 c/deg (see Fig. 5). This is an inherent problem if you want to use a small stimulus to selectively drive the M or P cells. As stated above, given the restricted frequency ranges over which the P (and especially the M) cells (see section Pulsed and Steady Pedestals), can be selectively driven, a narrowband stimulus has to be used.

Additionally, the size of the Gabor has to be such that it contains a whole number of cycles of the sinewave. A complete cycle of the sinewave results in an equal amount of luminance modulation both above and below the background (mean) luminance level. An incomplete cycle results in a luminance offset in the Gabor (either greater or lower than the background luminance, depending on the phase of the Gabor). A Gabor that contains a mean-luminance offset also has a broader spatial-frequency offset than one that does not (Bracewell, 1978).

Note that another way to produce stimuli that have narrow spatial-frequency bandwidths is to spatially filter broadband stimuli. This involves either convolving the image with a spatial filter that has the required spatial-frequency tuning or filtering via a Fourier transform of the image (J. C. Badcock, Whitworth, Badcock, & Lovegrove, 1990).

Finally, as highlighted previously, if the waveform of the luminance sinewave is distorted, then the stimulus is not a pure sinewave, and so the spatial-frequency bandwidth will be broadened. This means that great care needs to be taken in generating the stimulus. This places a number of demands on both the computer monitor (i.e., the screen) being used and how it is controlled. Specifically, one needs to use a monitor that does not add luminance distortions across its extent, and which also has good temporal resolution. This latter requirement typically means that a cathode-ray-tube monitor needs to be used. Additionally, these images are typically generated via computer code in which a mathematical array stores the Gabor profile and then displays that as an image on the screen by converting those values to luminance intensities via a look-up-table (LUT) conversion. It is vital that the LUT used is linear. That is, a plot of the luminance value generated versus LUT value results in a straight line. For an 8-bit LUT, this would be for values from 0 to 255. Having a linear LUT means that a sinewave variation in the LUT values around the midpoint (128) of that LUT would result in balanced luminance increments and decrements (i.e., an actual sinewave variation in luminance). Unfortunately, the variation in luminance as a function of LUT value is not linear for most monitors, which, if used without correction, would result in a distorted luminance profile. Typically, the increase in luminance above the mean LUT value is greater than the decrease below it, meaning that the light increments of the bright regions of the “sinewave” would be more extreme changes than the dark regions. The relationship between LUT value and luminance is typically well modeled by a gamma function, hence a gamma-corrected LUT needs to be used to avoid this distortion occurring, which involves using a photometer to create an inverse-gamma function to calibrate the monitor (Metha, Vingrys, & Badcock, 1993).

In spite of these limitations, a number of studies have used low versus high spatial-frequency defined stimuli to selectively drive the M and P pathways, respectively, and have found differential results for the different spatial frequencies (Abrams & Weidler, 2014; Goodhew et al., 2017; Goodhew & Clarke, 2016). For example, a manipulation designed to decrease the size of the attended region improved orientation-discrimination performance when high-spatial-frequency Gabors were used, but not low-spatial-frequency Gabors (Goodhew et al., 2017; but also see Lawrence et al., 2020). These studies suggest that it is possible to selectively drive the M and P pathways via low and high spatial-frequency stimuli. As we will see in the next section, the strongest evidence for low (i.e., 2 c/deg or less) spatial frequencies being selective processed by M cells comes from the human psychophysical studies that have used a luminance pedestals.

### Pulsed and steady pedestals

Pokorny and Smith (Pokorny, 2011; Pokorny & Smith, 1997) have developed another way to determine the role of M and/or P cells in a given task. This is a masking technique that, for M cells, is based on the selective sensitivity of the M cells to onset and/or offset luminance transients and involves presenting the stimulus on the luminance pedestal. This pedestal is a region that has different (higher or lower) luminance than the background, and its magnitude is varied in one of two ways in order to either drive or not drive the M cells. In the pulsed-pedestal condition, the luminance of the pedestal is pulsed on with the brief target stimulus so as to drive the M cells, while in the steady-state-pedestal condition, the luminance remains constant so the M cells are not driven by the pedestal, but the P cells are slowly adapted. The strong response of the M cells to the pulsed pedestal means that they are unable to give a selective response to any stimulus placed on that pedestal (i.e., their response is masked because of saturation in their response to higher contrasts). Hence, the P cells will mediate performance in the pulsed-pedestal condition. In the steady-pedestal condition, the P cell sensitivity is reduced because of adaptation arising from the continuous presentation of the pedestal and the brief target, with onset and offset transients, strongly drives the M cells. This means that by comparing performance between the two conditions, the relative roles of the M and P cells in a particular task can be determined (Goodhew, Boal, & Edwards, 2014; McKendrick & Badcock, 2003; McKendrick, Badcock, & Morgan, 2004; Pokorny, 2011; Pokorny & Smith, 1997; Yeshurun & Sabo, 2012).

This pedestal approach can also be combined with the use of a raised-cosine temporal window to further impair the ability of the M system to selectively respond to the stimuli, thus allowing for the further investigation of the sensitivity of the P system to the stimuli. This approach has provided good evidence that M cells mediate spatial frequencies below 2 c/deg, at least at threshold-contrast levels (Leonova et al., 2003).

### Motion and color defined stimuli

Motion stimuli can also be used to try to selectively drive the M system, though the P system also appears to play a role in motion processing at low speeds (i.e., low temporal frequencies; Merigan, Byrne, et al., 1991a), so if this technique is used, high-speed (greater than around 10^o^/s) stimuli should be used. Note that the ability to use color information to extract motion is consistent with P input into the motion system (Cropper, 2006; Edwards & Badcock, 1996).

Finally, stimuli defined purely by color could, in theory, be used to selectively drive P cells—that is, isoluminant stimuli in which the aim is to use stimuli that differ from the background in terms of color, but not luminance (e.g., a red square on a green background, with the red and green having the same luminance). However, in practice, it is not possible to achieve isoluminance at every stimulus location, especially for large stimuli, given that perceived luminance depends on the response of the long and medium wavelength-sensitive cones, and the ratio of these cones varies across the retina and their adaptation state changes quickly when presented with colored stimuli. Additionally, chromatic aberration occurs at the colored border of the stimulus, which can introduce a luminance border. All of this means that, while local luminance differences can be minimized in these chromatic stimuli, and this can dramatically change the percept (Cavanagh, Tyler, & Favreau, 1984; C. Lu & Fender, 1972; Ramachandran & Gregory, 1978), they cannot be fully removed, thus a stimulus needs to be used in which luminance information is present, but it cannot be used to process the signal. One way to do this is to have a mismatch in the polarity of the luminance information that needs to be combined. That is, for example, in a motion stimulus, alternate the luminance polarity as image moves (e.g., have a moving dot go from light; i.e., its luminance is above the background luminance) to dark (luminance below the background luminance) or to add dynamic luminance noise that is uncorrelated with the chromatic signal in order to mask any residual luminance components. Or, with a stereoscopic image, have the image to be matched across the two eyes be defined by opposite luminance polarities (light image in one eye and dark in the other; Edwards & Badcock, 1996; Stuart, Edwards, & Cook, 1992). Another way of keeping luminance information in the stimulus but arranging it such that it cannot be used to do the task is to add random variation to the luminance signal. This is how an Ishihara color test is designed. The number and background are composed of small subelements, and the luminance of these elements vary in a random manner, but they contain a consistent color difference.

### Selectively tapping the dorsal and ventral pathways

While it was initially thought that the segregation between the M and P pathways at the dLGN level was maintained in the dorsal and ventral cortical pathways, this has proven to be not the case (J. H. Maunsell & Newsome, 1987). However, the dorsal pathway receives more substantial input from the M pathway and the ventral from the P pathway (M. S. Livingstone & Hubel, 1987). Thus, a number of studies have sought to selectively tap the dorsal and ventral pathways in order to investigate the role of M and P dominated inputs at the cortical level, for example. Effective stimuli to achieve this aim are global-motion and Glass-pattern stimuli. The processing of global-motion stimuli has been linked to cortical area V5/MT in the dorsal pathway (Newsome & Pare, 1988; Newsome, Salzman, Murasugi, & Britten, 1990; Salzman, Britten, & Newsome, 1990) and Glass stimuli (Glass, 1969) to areas in the ventral pathway (Gallant, Shoup, & Mazer, 2000; Pei, Pettet, Vildavski, & Norcia, 2005; Wilson & Wilkinson, 2015).

Both of these stimuli have the same logic in that they consist of discrete signal and noise elements. The global-motion stimulus consists of a number of moving dots, with signal elements moving in the same (i.e., global) motion direction while the noise elements move in random directions (that do not include the global direction; Newsome & Pare, 1988). The Glass stimulus consists of a number of static dot dipoles—that is, two dots in close proximity, with signal elements arranged such that if there was a line joining them, they would all have orientations consistent with a common global pattern (linear, circular or radial), while the noise elements are orientated randomly (Dickinson & Badcock, 2007; Glass, 1969). This means that both stimuli involve extracting signal from noise, and the metric is the same for both of them: the coherence level required to perceive the signal. In addition, experimental paradigms have been developed that makes it possible to use these stimuli to tap either the V1 mediated extraction of the signal or the global-pooling stage (Edwards & Badcock, 1994, 1995, 1996). Note also, a Gabor version of the global-motion stimulus has been developed to enable spatial frequency to be controlled and manipulated (Amano, Edwards, Badcock, & Nishida, 2009a, 2009b), and a similar Gabor-based stimulus has been used to examine the global processing of form (Bowden, Dickinson, Fox, & Badcock, 2015; Tan, Bowden, Dickinson, & Badcock, 2015).

### Reliability considerations

When any of the above tasks are used in studies that aim to explore individual differences in M or P processing, the reliability of people’s performance on those tasks needs to be considered. This is because the ability to find correlations between performance on these tasks—for example, global-motion performance, and, for example, their score on a measure of schizophrenia—depends on the reliability of these measures in isolation. For a more detailed discussion of these issues, see Goodhew and Edwards (2019) and Hedge, Powell, and Sumner (2018).

### Conclusions

From our review of the literature, it is clear that while there is substantial overlap in the tuning properties of the M and P cells, it is the case that there are differences in their sensitivity to some visual properties that they preferentially process. Thus, it is possible to design and use visual stimuli to preferentially drive the cells, but, given that tunings overlap, great care needs to be taken in the selection and design of such stimuli. In these considerations, the entire parameter space of the stimuli needs to be considered. In particular, when the aim is to observe the influence of the M cells, the stimuli should be defined by a combination of high temporal frequencies, low spatial frequencies, and low luminance contrast. Conversely, when the aim is to more clearly observe the influence of the P cells, the stimuli should be defined by low temporal frequencies and high spatial frequencies. Whether a high (greater than 10%) luminance contrast is required, if the spatial and temporal frequency of the stimuli are appropriate, is debatable. The experimental paradigms that make it easiest to achieve these stimulus combinations are temporal and spatial acuity tasks. These stimulus parameter combinations can also be achieved by using Gabor stimuli in, for example, an orientation discrimination task. However, with such stimuli, great care needs to be taken to ensure that the temporal and spatial frequency bandwidths of the stimuli are sufficiently narrowband.
